# Epidemiological and clinical characteristics of Dengue virus outbreaks in two regions of China, 2014 – 2015

**DOI:** 10.1371/journal.pone.0213353

**Published:** 2019-03-05

**Authors:** Jiaqi Cao, Hong Deng, Lei Ye, Xuezheng Ma, Shuru Chen, Xiaohong Sun, Xuemin Wu, Tao Yan, Liping Zhang, Lijuan Liu, Lili Li, Wuping Li, Kongxin Hu

**Affiliations:** 1 NHC Key Laboratory of Systems Biology of Pathogens, Institute of Pathogen Biology, Chinese Academy of Medical Sciences & Peking Union Medical College, Beijing, P. R. China; 2 The Third Affiliated Hospital of Sun Yat-Sen University, Guangzhou, China; 3 Institute of Health Quarantine, Chinese Academy of Inspection and Quarantine, Beijing, China; Institut Pasteur, FRANCE

## Abstract

Dengue virus (DENV), a single-stranded RNA virus and Flaviviridae family member, is transmitted by *Aedes aegypti* and *Aedes albopictus* mosquitoes. DENV causes dengue fever, which may progress to severe dengue. Hospital-based surveillance was performed in two Chinese regions, Guangzhou and Xishuangbanna, during the dengue epidemics in 2014 and 2015, respectively. Acute-phase serum was obtained from 133 patients with suspected dengue infections during the peak season for dengue cases. Viremia levels, virus sero-positivity, serotype distribution, infection type, clinical manifestations and virus phylogenetics were investigated. Of the 112 DENV-confirmed cases, 92(82.14%) were IgM antibody-positive for DENV, and 69(51.88%) were positive for DENV RNA. From these cases, 47(41.96%) were classified as primary infections, 39(34.82%) as secondary infections and 26 (23.21%) as undetermined infections. The viremia levels were negatively correlated with IgM presence, but had no relationship with the infection type. DENV-1 genotype V dominated in Guangzhou, whereas the DENV-2 Cosmopolitan genotype dominated in Xishuangbanna, where fewer DENV-1 genotype I cases occurred. DENV-2 is associated with severe dengue illness with more serious clinical issues. The strains isolated during 2014–2015 are closely related to the isolates obtained from other Chinese regions and to those isolated recently in Southeast Asian countries. Our results indicate that DENV is no longer an imported virus and is now endemic in China. An extensive seroepidemiological study of DENV and the implementation of vector control measures against it are now warranted in China.

## Introduction

Dengue virus (DENV), a *Flavivirus* genus member of the *Flaviviridae* family, is transmitted by *Aedes aegypti* and *Aedes albopictus* mosquitoes. The virus is endemic in many tropical and subtropical countries where it is associated with outbreaks of dengue disease. Globally, approximately 390 million people are estimated to be infected with DENV each year, and disease manifestations occur in around 96 million people [1]. The clinical signs and symptoms of DENV infections can manifest themselves as mild fever, hemorrhagic fever, or fatal shock syndrome, or infections can be asymptomatic [2,3]. Dengue, the second most important vector-borne disease compared with malaria. Currently, the CYD-TDV vaccine (dengvaxia) is licensed since 2015 in 20 countries with low efficacy and increased risk of severe disease among naïve individuals [4,5,6,7,8,9], but there are no specific therapeutics available for it, and substantial vector control efforts have not prevented its rapid re-emergence and global spread, a situation that poses a great threat to human health.

The DENV genome comprises a single strand of positive-sense RNA encoding three structural and seven nonstructural proteins. DENV is divided into four serotypes (DENV-1, -2, -3 and -4), each of which can be sub-divided into several genotypes based on the envelope gene, which confers partial cross-protective immunity against the other serotypes in humans [10]. DENV-1 is sub-divided into five genotypes: I, II, III, IV and V [11]. DENV-2 is classified as six genotypes: Asian I, Asian II, Southeast Asian/American, Cosmopolitan, American and sylvatic [12,13,14]. DENV-3 comprises five genotypes including I–V [15]and DENV-4 has four genotypes consisting of I–III and the sylvatic genotype [16].

The geographic distribution of DENV infection on mainland China based on a number of large dengue outbreaks with serious consequences have been reported shows that the epidemic was caused by an imported virus, because this virus has not yet been confirmed to be endemic in this country [17]. The first outbreak of dengue after World War II in China occurred in Guangdong Province in 1978 [18]. Thereafter, the two most severe outbreaks were in 1980 and 1986 in Hainan Province, and resulted in more than 600,000 cases and 475 deaths [19]. Since then, dengue epidemics have been recorded sequentially in Guangdong, Guangxi, Fujian and the relatively northern and western regions of China including Zhejiang and Yunnan Provinces, with shorter epidemic intervals [20,21,22,23]. In recent years, Guangdong and Yunnan Provinces have experienced the highest incidence of dengue epidemics, with cases being reported every year since 1997 [24,25,26]. The characteristics of the temporal distribution in mainland China indicate that the disease occurs sporadically from January to May, whereas June to December is recognized as being the prevalent period [27]. Four serotypes, DENV-1, 2, 3, and 4, have been identified and remain prevalent in China.

In the present study, we analyzed the envelope gene profile of DENV and characterized the sero-prevalence, serotype distribution, type of infection, clinical manifestations and phylogenetic characteristics of outbreaks caused by this virus in Guangzhou and Xishuangbanna during 2014 and 2015.

## Materials and methods

### Ethics statement

This study was approved by the Ethics Committee of the Institute of Health Quarantine, Chinese Academy of Inspection and Quarantine with approval number IEC/2013/10. Clinical data and blood samples were identifiable only by serial study numbers having been anonymized by de-linking study numbers from patients’identifying information in order to maintain confidentiality. The authors had no access individual patient information during or after collection.

### Specimen collections

**Fig 1:**
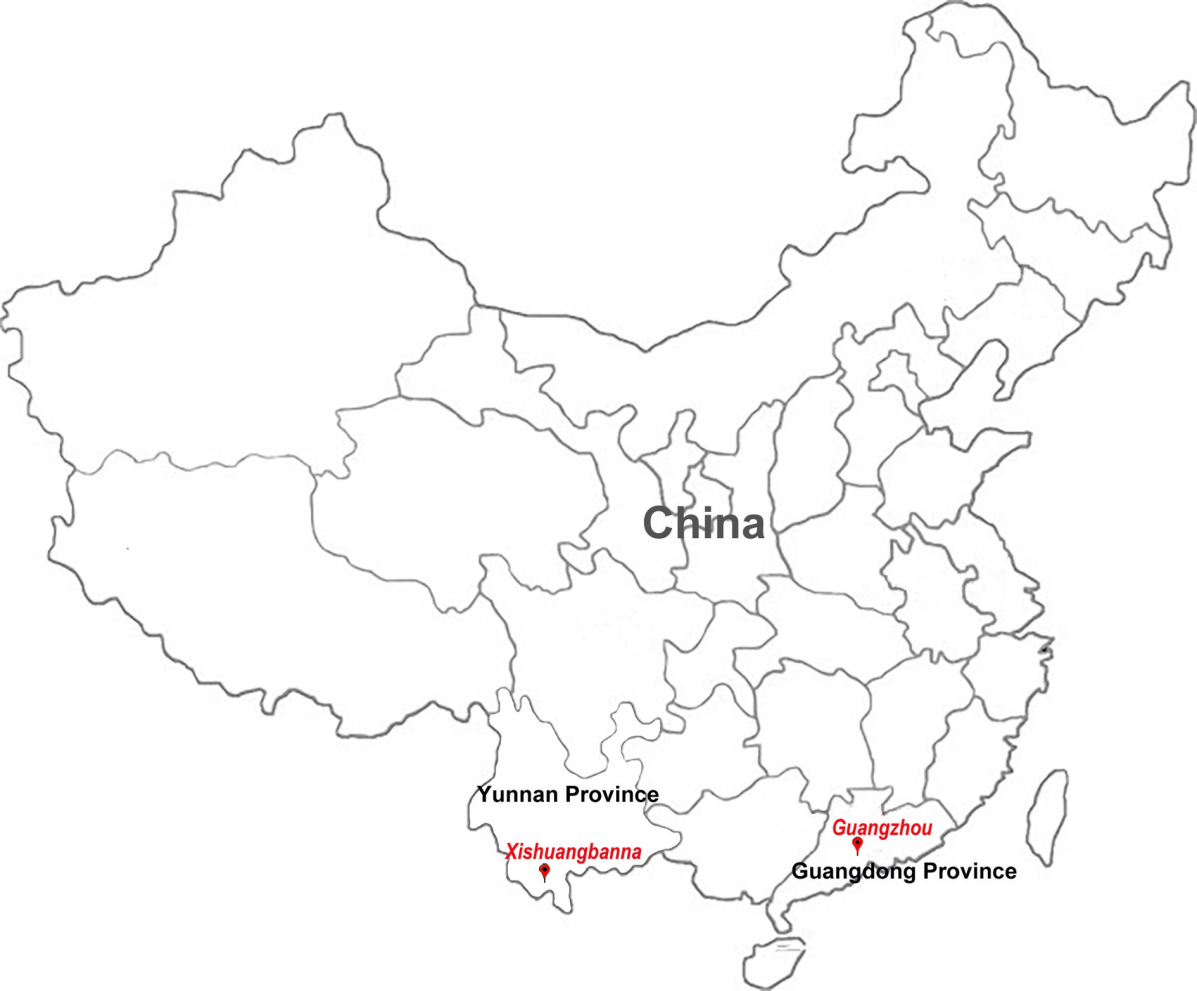
The location of the two study sites in China. Xishuangbanna, Yunnan province locate at southwest of China adjacent to Southeast Asian country and Guangzhou, Guangdong province locate at southern of China.

The selected study sites were Guangzhou in Guandong Province and Xishuangbanna in Yunnan Province where dengue epidemics occurred in 2014 and 2015 respectively. Their locations are shown in Fig 1. Yunnan Province in Southwest China has both tropical and subtropical regions and shares borders with South Asian countries (Vietnam, Laos, Myanmar) that are dengue endemic areas. Guangzhou is one of largest city with the highest population density in the world. It is the capital city of Guangdong Province in southern China and has a humid subtropical climate influenced by the Asian monsoon season. Patients were recruited from two hospitals in Xishuangbanna (Jinghong Farm Staff Hospital and Xishuangbanna Mental Health Center) and one hospital in Guangzhou (The Third Affiliated Hospital of SUN YAT-SEN University). During the DENV outbreaks, 105 whole blood specimens collected in Xishuangbanna (November 2015) and 28 collected in Guangzhou (October 2014) who presented with suspected dengue virus infection were screened for evidence of DENV infections from 1 to 14 days after presentation at the hospital. After the serum was separated from each peripheral blood specimen the samples were stored at –20°C until processing. Patients suspected of a having dengue infection, as determined by a fever greater than 38°C taken at axillary accompanied by at least one clinical sign or symptom of dengue fever such as headache, rash, arthralgia, retro-orbital pain, malaise, signs of dengue hemorrhagic fever or dengue shock syndrome were enrolled. A case of dengue was confirmed when a patient suspected of having the disease tested positive by at least one of the following diagnostic tests: NS1 antigen (Panbio Dengue Early ELISA kit), anti-dengue immunoglobulin M (IgM) (Panbio Dengue IgM Capture ELISA kit) or reverse-transcriptase real-time polymerase chain reaction (RT-PCR). The clinical features were obtained from the medical records of the patients. The laboratory tests, which included the white blood cell count, hematocrit, platelet count, and transaminase levels, were analyzed chronologically from the onset of the clinical signs and symptoms for each patient.

### Viral RNA extraction, RT-PCR serotyping, copy number assessment, and DNA sequencing

Total RNA was extracted from the serum samples using the Qiagen RNeasy Mini kit (Qiagen). Virus serotyping and genome copy numbers for each sample were determined using the DENV general-type real-time RT-PCR Liferiver kit (Shanghai ZJ Bio-Tech Co, China). Briefly, total RNA was extracted from 140μl of human serum using a Qiagen RNeasy Mini kit (Qiagen), as per manufacturer's protocol. RNA was eluted in 50μl of AVE buffer provided with the kit. 5μl of RNA was then added to the RT-PCR mix. The kits can detect 10^3^ copy/ml. Gene-specific primers were designed to amplify and sequence the envelope gene sequence from the virus. The D1-863F/2462R primer set (5’-TGCCATAGGAACATCCATCAC-3’ and 5’-TCCCAATGGCTGCTGATAGTC-3’) was used for RT-PCR amplification of the DENV-1 sequences and D2-556F/2160R (5’-GACCTTGGTGARTTGTGTGAAG-3’ and 5’-CARTCTTGTTACTGAGCGGA-3’) was used for RT-PCR amplification of the DENV-2 sequences. Both primer sets were also used for DNA sequencing of the viruses. The PrimeScript RT Reagent kit (TaKaRa) was used for reverse transcription and the PrimeSTAR GXL DNA Polymerase (TaKaRa) was used for PCR. Reverse transcription was conducted at 37°C for 15 min, 85°C for 5 s, followed by PCR amplification at 94°C for 2 min, 35 cycles of 94°C for 30 s, 55°C for 30 s, and 72°C for 2 min, with a final extension of 72°C for 5 min.

### Serological tests

Serum samples from the suspected dengue patients, collected during the 1–14-day after onset of fever, were subjected to serological testing and dengue antigen detection. The preliminary screening for the presence of DENV NS1 protein in patients’ sera was performed using the Panbio Dengue Early ELISA kit (Alere, Brisbane, Australia) to detect the NS1 according to the manufacturer's instructions. A positive NS1 result (> 11 Panbio units) is considered to be indicative of an active primary or secondary dengue infection. Anti-dengue IgG and IgM detection was performed using the Panbio Dengue IgM and IgG Capture ELISA kit, which was also used to determine the infection status (primary or secondary infection) of a sample according to manufacturer’s protocol. In essence, a positive IgM result (> 11 Panbio units) indicated an active primary or secondary infection, if a positive IgG result (> 22 Panbio units) was defined as an active secondary infection, which could be accompanied by elevated IgM levels.

### Gene sequencing and phylogenetic analysis

The whole envelope protein gene from all the isolated viral strains was PCR-amplified using gene-specific primer sets. Phylogenetic trees were constructed based on the full envelope gene region of the viral strains isolated in this study and from those previously isolated in China and its neighboring countries, and from other regions of the world. The sequences obtained were aligned with other DENV sequences available in the GenBank database (www.ncbi.nlm.nih.gov). To identify the genotypes from the sample sequences, maximum-likelihood trees were constructed in MEGA version 6.0 [28] using 50 sequences (S2 Table) for DENV-1 [29] and 55 sequences (S3 Table) for DENV-2, all of which belong to different genotypes and come from different samples. Bootstrap values were set at 1000 reiterations.

### Statistical analysis

Data analysis was performed using IBM SPSS software version 21.0 [30]. Mean values between two groups were compared using the unpaired Student’s t test. Mean values among the groups were compared using a one-way analysis of variance test. Pearson’s Chi-square or Fisher’s exact tests were used to compare univariate categorical data. *P* values of <0.05 were assumed to be statistically significant.

## Results

### Characteristics of the laboratory-confirmed dengue cases

In total, 133 individuals with suspected dengue virus infection who each provided a single acute-phase serum sample were investigated in the two Chinese study sites. The median ages of the patients in Xishuangbanna and Guangzhou were 37 (range, 11–78) and 42 (range, 22–81), respectively. The age distributions of the patients showed that the most frequent age groups in Xishuangbanna and Guangdong were 20–29 years and >70 years, respectively. Of the 112 confirmed dengue cases, 92 patients (82.14%) were DENV IgM positive whereas 74 of the 94 available serum samples (78.72%) showed DENV-NS1 antigen positive (S1 Fig). Based on the results of the anti-dengue IgG testing, 47 (41.96%) of the cases were classified as having primary infections, 39 (34.82%) were secondary infections, whereas 26 (23.21%) had undetermined infections. The median age was 36 years (range, 11–75) for the patients with primary infections and 38 years (range, 18–81) for those with secondary infections.

### DENV RNA prevalence, viremia levels, and antibodies in the suspected cases

**Fig 2:**
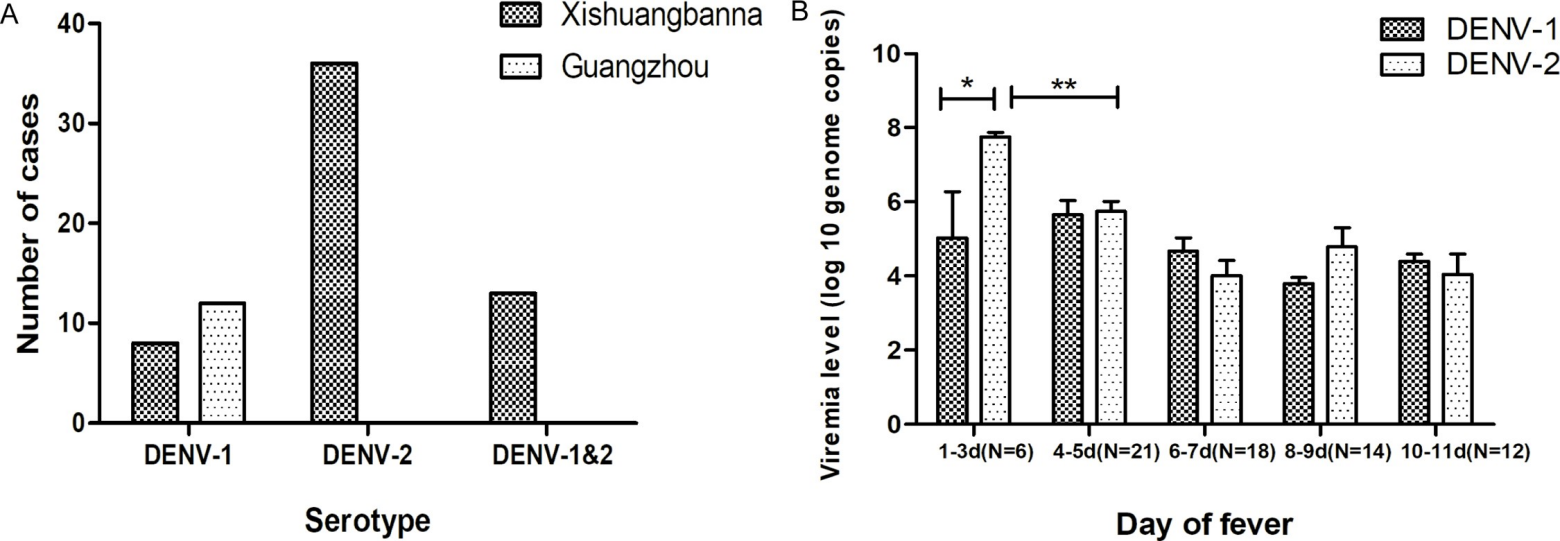
Serotyping and quantitating of DENV in the two study areas. (A) A comparison of the serotypes by RT-qPCR in the two study regions and (B) titering according to the onset day of fever in patients from which DENV RNA was isolated.

Serotyping RT-qPCR was performed to estimate the prevalence of DENV RNA in the samples. As shown in Fig 2A, of the 133 serum samples, 69 (51.88%) were positive for reverse-transcribed DENV DNA. In Xishuangbanna, eight samples (14.04%) from the isolates were typed as DENV-1 and 36 (63.16%) of them as DENV-2. There were 13 instances (22.80%) of mixed infections with two serotypes (DENV-1 and -2). In contrast, in Guangzhou, all 12 strains were DENV-1. RT-qPCR was used to estimate the viral titer corresponding to the day of fever when the DENV RNA was isolated from each patient. As shown in Fig 2B, the viremia levels in the patients infected with DENV-2 were significantly higher than those from the patients infected with DENV-1 on days 1 to 3 (p<0.05) after onset of fever. Then, the viremia levels were demonstrated a declining trend of both serotypes with no significant difference.

**Fig 3:**
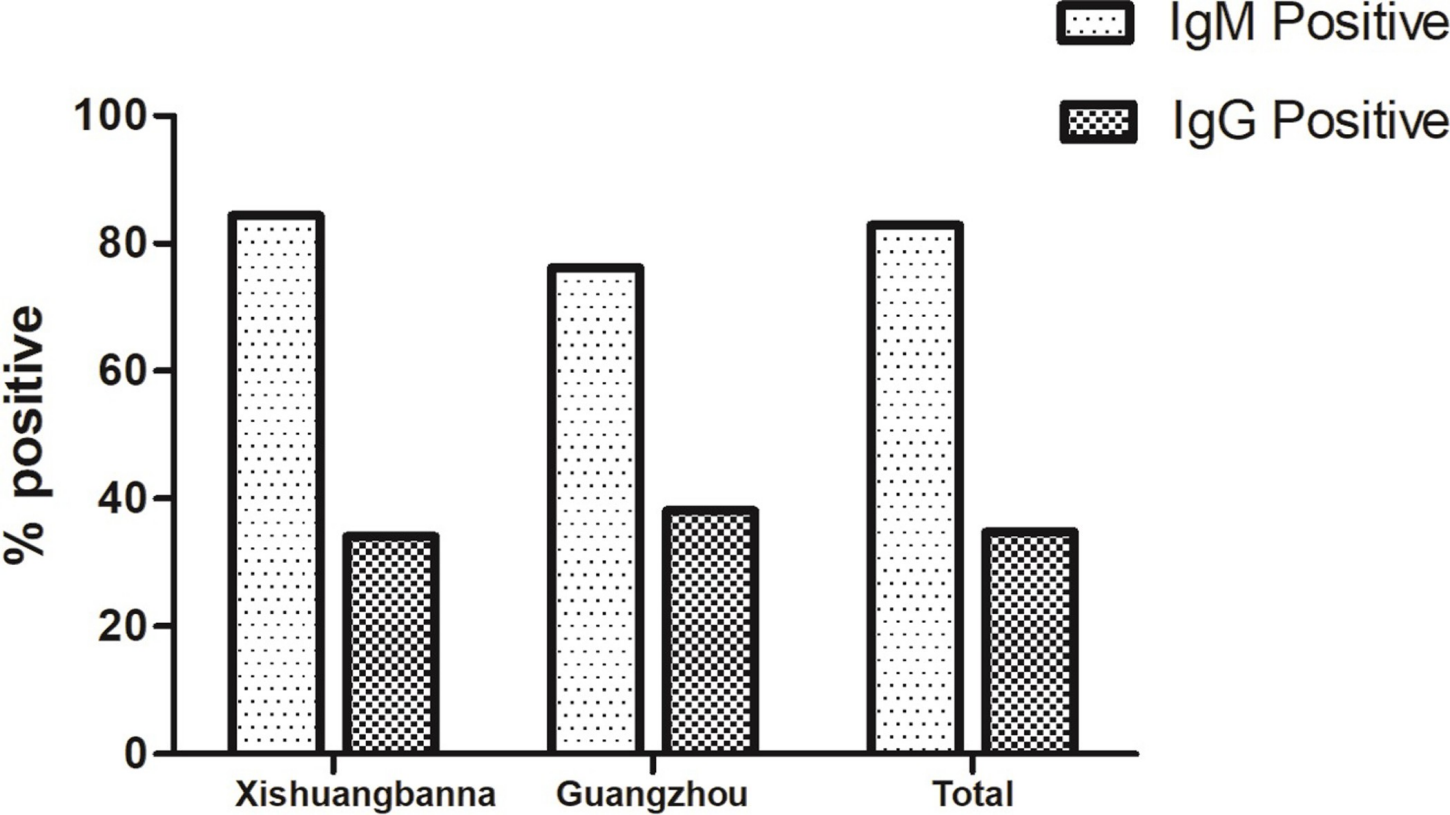
Seroprevalence of DENV-IgG and seroincidence of DENV-IgM antibodies in the patients from Guangzhou and Xishuangbanna of China.

**Fig 4:**
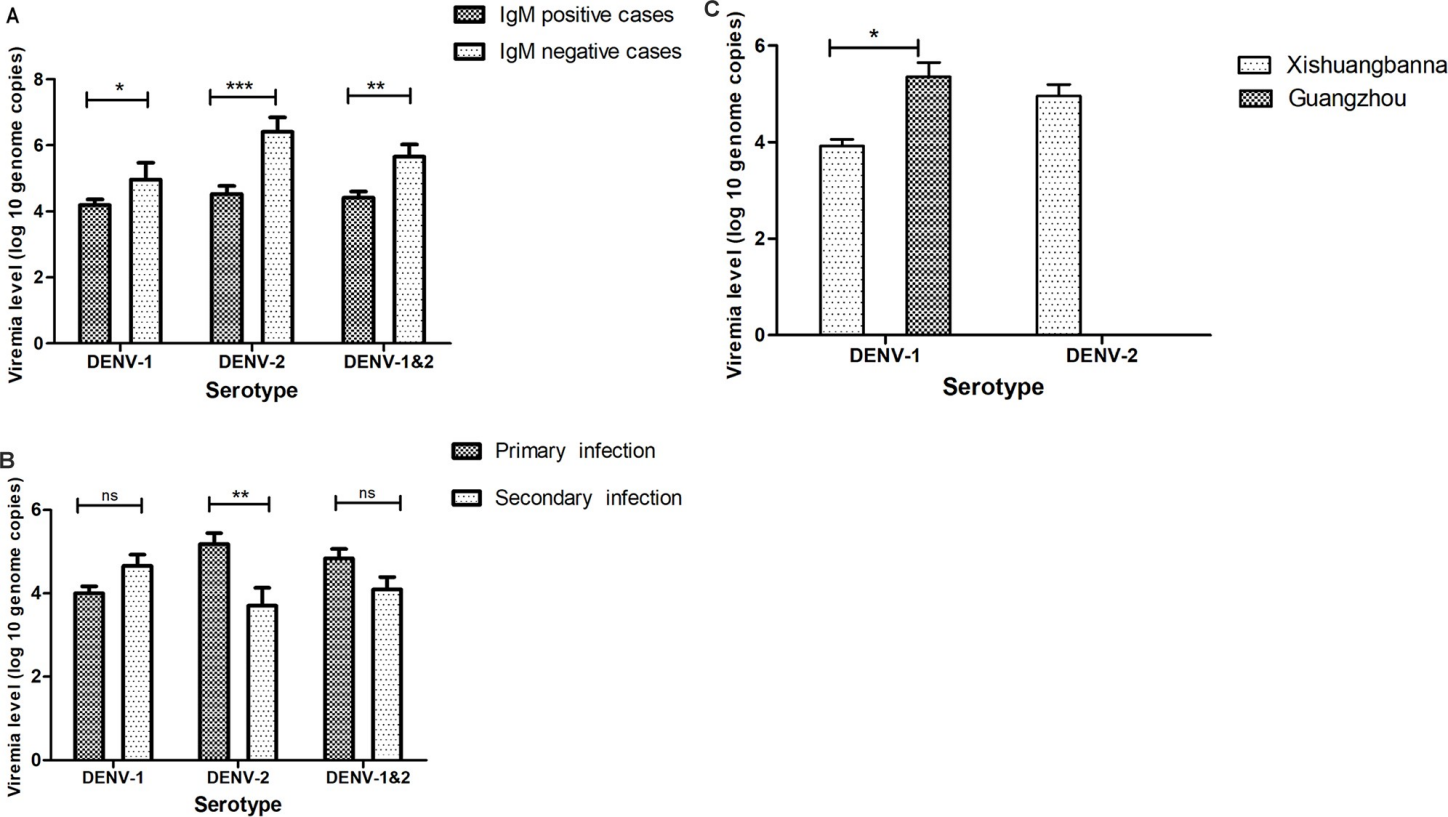
Comparison of viremia levels of dengue patients as estimated by RT-qPCR. (A) in DENV IgM positive vs DENV IgM negative patients. (B) and in primary vs secondary DENV infections. (C) in Guangzhou and Xishuangbanna regions.

The serological characteristics of the serum samples were also examined using an enzyme-linked immunosorbent assay. As shown in Fig 3, recent DENV infection rates in Xishuangbanna (84.4%) and Guangzhou (76.2%) detected by DENV IgM were much higher than past DENV infection rates in these two study areas [Xishuangbanna (34.10%) and Guangzhou (38.1%)] detected by DENV-IgG. The viremia levels were significantly higher in the DENV IgM seronegative samples than those for the DENV IgM positive sera samples (p <0.01) (Fig 4A).

To find out the relationship of viral load with IgM positivity, infection type and serotypes of DENV in different study areas, we analyzed in Fig 4. The results revealed the IgM positive cases is always associated with lower virus load (Fig 4A) and patients with primary infection with DENV-2 had significantly higher viral titer than those with secondary infection with DENV-2 (P<0.01) (Fig 4B). However, such a difference was not observed in the patients with DENV-1 infections or for those with mixed DENV-1+DENV-2 infections(P>0.05)(Fig 4B). In addition, the mean viral titer for DENV-1 among patients in Guangzhou was one log higher than those in Xishuangbanna(p = 0.012)(Fig 4C).

### Associations among clinical and laboratory features and infection types

**Fig 5:**
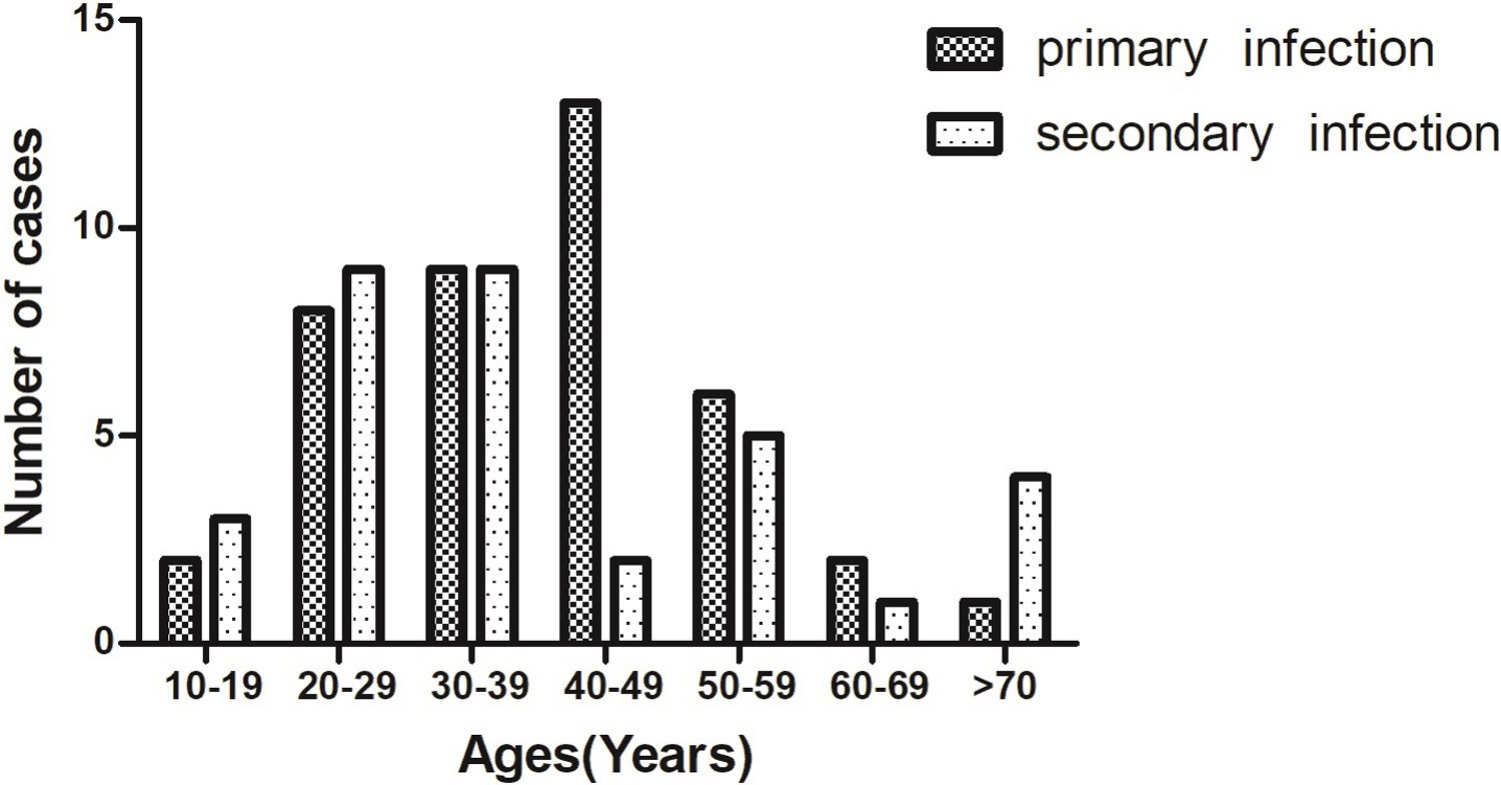
Distributions of primary and secondary DENV infections among patients in different age groups.

**Fig 6:**
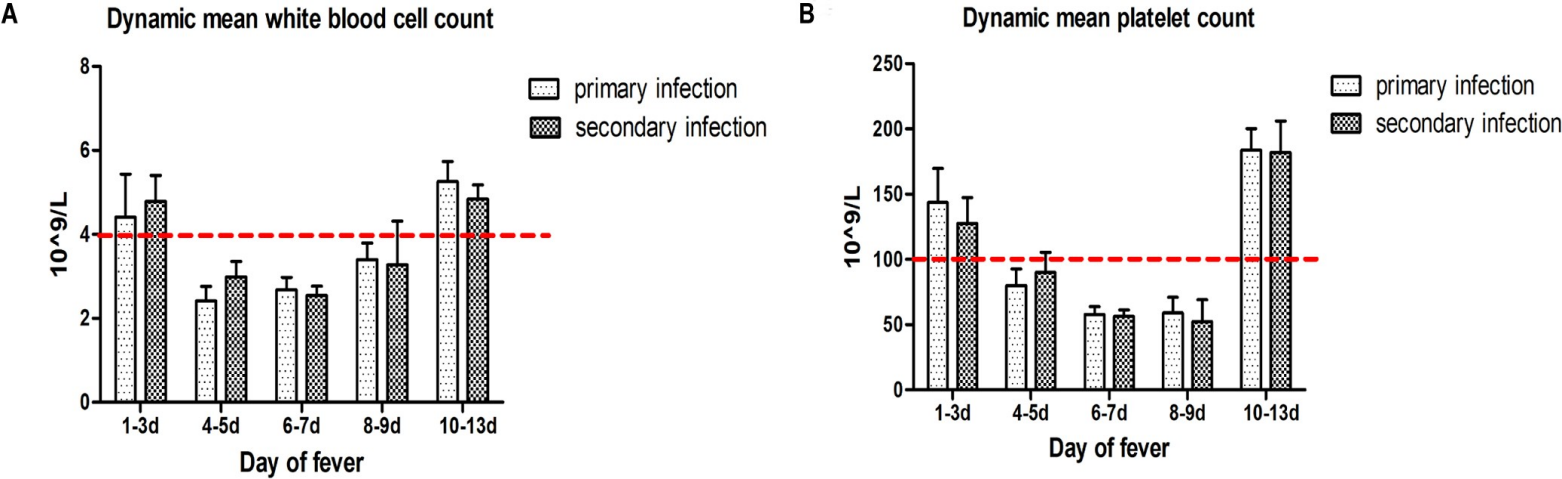
Mean values of (A) white blood cell counts and (B) platelet counts in DENV positive patients with primary or secondary DENV infection at different onset days after fever.

A comparative analysis was conducted on the demographic data, laboratory parameters, infection types (serotypes of DENV, primary or secondary DENV infections), and the clinical manifestations of the disease. As shown in Fig 5, the patients with primary infections had a median age of 36 years (range, 11–75), while the median age for secondary infections was 38 years (range, 18–81). The age distributions showed that the commonest age group for primary and secondary DENV infection cases were 40–49 years and 20–39 years, respectively. The relationships among the clinical and laboratory parameters and the infection serotypes are shown in Table 1. There were significant differences between the clinical features and the serotypes of DENV infected, even though there were no differences in age, gender, occupation for patients infected with DENV-1 versus DENV-2 (Table 1). Those patients who were infected with DENV-2 or with a mixed infection containing this serotype had significantly higher rates of headache, anorexia and nausea, vomiting and debilitation and significantly higher rates of abnormal liver function values. There were no significant differences among white blood cell count, platelet count and infection type (Fig 6A and 6B), but a decline was observed from days four to nine after onset of fever and then recovered to the normal level at ten to thirteen days. No significant associations among the demographics and clinical signs for primary versus secondary infections were noted, except that a higher bleeding rate was observed for the secondary infections (S1 Table).

**Table 1:** Clinical and laboratory outcome measures for patients with DENV-1, DENV-2 vs DENV-1 mixed with DENV-2 infection.

Serotype	Total	DENV-1	DENV-2	DENV-1&2	p
(N = 43)	(N = 43)	(N = 13)	(N = 19)	(N = 11)	
**Age in years, mean(SD)**	43.7(18.29)	51.5(21.13)	42.2(18.19)	36.9(13.10)	0.138
**Gender, % female**	25(58.14%)	7 (53.85%)	12(63.16%)	6(54.55%)	0.95
**Fever in the last 7days**	39(90.70%)	10(76.92%)	18(94.74%)	11(100%)	0.22
**Occupation (%)**					
**Unemployed/ retired**	15(34.88%)	4(30.77%)	9 (47.37%)	2(18.18%)	ND
**Laborer**	11(25.58%)	2(15.38%)	4 (21.05%)	5(45.45%)	ND
**Merchant/office**	12(27.91%)	4(30.77%)	5 (26.32%)	3(27.27%)	ND
**Other**	5(11.63%)	3(23.08%)	1 (5.26%)	1(9.09%)	ND
**Clinical sign and symptoms (%)**					
**Headache**	28(65.12%)	4 (30.77%)	15(78.95%)	9(81.82%)	**0.016**
**Cough**	7(16.28%)	1 (7.69%)	5 (26.32%)	1(9.09%)	0.471
**Anorexia and nausea**	25(58.14%)	1 (7.69%)	15(78.95%)	9(81.82%)	**0**
**Muscle/joint pain**	31(72.09%)	10(76.92%)	14(73.68%)	7(63.64%)	0.904
**Rash**	7(16.28%)	2 (15.38%)	4 (21.05%)	1(9.09%)	0.863
**Bleeding**	7(16.28%)	6 (46.15%)	0	1(9.09%)	**0.006**
**Vomiting**	11(25.58%)	0	7 (36.84%)	4(36.36%)	0.093
**Debilitation**	23(53.49%)	3 (23.08%)	13(68.42%)	7(63.64%)	0.072
**Abdominal pain**	2(4.65%)	1 (7.69%)	1 (5.26%)	0	0.844
**Diarrhea**	5(11.63%)	2 (15.38%)	2 (10.53%)	1(9.09%)	0.966
**Main laboratory results during the course of the disease (%)**					
**Thrombocytopenia(<100×10**^**9**^**/L)**	30(69.77%)	8 (61.54%)	14(73.68%)	8(72.73%)	0.896
**Leukopenia(<4×10**^**9**^**/L)**	36(83.72%)	11(84.62%)	16(84.21%)	9(81.82%)	0.998
**ALT(>40U/L)**	37(86.05%)	8 (61.54%)	18(94.74%)	11(100%)	**0.024**
**AST(>40U/L)**	38(88.37%)	9 (69.23%)	18(94.74%)	11(100%)	0.077

*ND*: Nodate

### Phylogenetic analysis

The envelope gene sequences from 19 DENV isolates were obtained from samples from the study areas in Guangzhou and Xishuangbanna. Among them, six isolates belonged to serotype 1 (four from Guangzhou and two from Xishuangbanna), while the other 13 isolates belonging to serotype 2 all came from Xishuangbanna.

**Fig 7:**
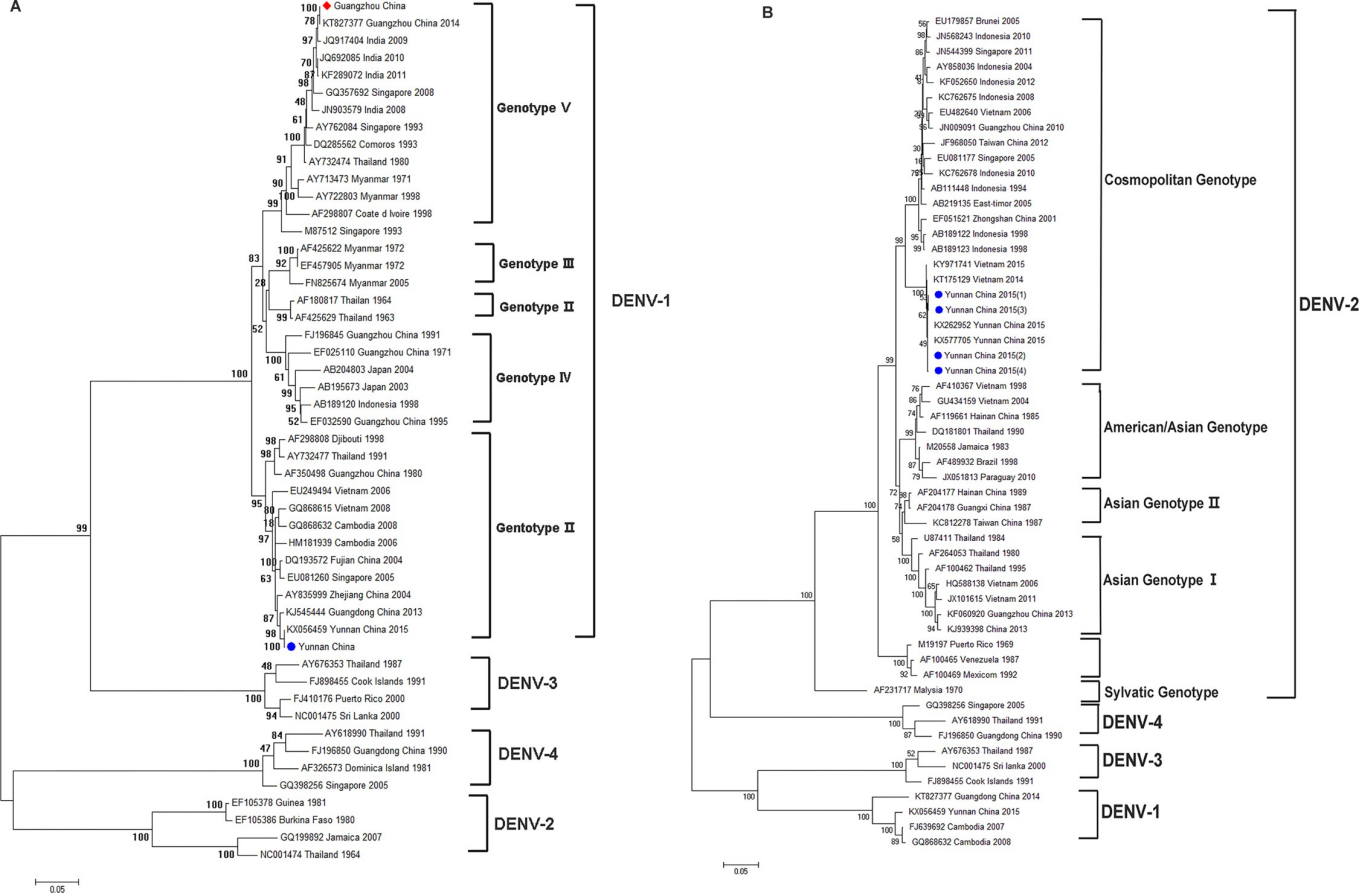
DENV phylogenetic trees. (A) The DENV-1 tree was constructed using the whole nucleotide envelope protein gene sequences from DENV-1 and shows the relationships of 50 strains from different sources including 10 DENV-1 strains isolated in China. (B) The DENV-2 tree was constructed using the whole nucleotide envelope protein gene sequences from DENV-2 and shows the relationships of 55 strains from different sources including 12 DENV-2 strains isolated in China. The sequences of this study were indicated as red diamonds (Guangzhou) and blue circles (Xishuangbanna).

A phylogenetic tree representing the full coding region of the envelope protein from the DENV-1 strains was constructed. As shown in Fig 7A, four strains from Guangzhou belong to genotype V and two isolates from Xishuangbanna sub-cluster with genotype I. These four strains from Guangzhou are 100% identical to the KT827377 strain obtained from Guangdong in 2014 and are closely related to isolates obtained in India from 2009–2011, suggesting that this isolate was possibly imported into China. The two isolates from Xishuangbanna each display high similarity to the isolates sporadically found in Zhejiang and Guangdong Province and are identical to the KX056459 isolate from Yunnan.

All 13 DENV-2 isolates that came from Xishuangbanna belong to the Cosmopolitan genotype and formed two clades in the phylogenetic tree (Fig 7B). Most of the strains are highly similar to the strains circulating in Vietnam and the Chinese strains, and share 98.8% nucleotide identity with strains previously isolated in Yunnan. To identify amino acid variation that could potentially influence the DENV-2 virulence profile of DENV-2, we compared the complete envelope protein gene sequence with KX577705, which was isolated in Yunnan in 2015. One amino acid substitution (D215N) in 5 of 13 isolates was observed (S2 Fig), but further studies will be needed to clarify whether a connection exists between virulence and genetic diversity in this virus.

## Discussion

Dengue fever is an acute, emerging infectious disease caused by DENV, which is transmitted by *Aedes aegypti* and *Aedes albopictus* mosquitoes causing around 50–100 million disease cases every year, and with its associated high morbidity and mortality, especially in developing countries, poses a great threat to public health worldwide [31]. The case fatality rate for untreated dengue patients was around 20%, but with early diagnosis, better clinical management and improved fluid replacement it has fallen to less than 1% [32]. Over the last four decades, three large dengue outbreaks resulting in around 0.7 million cases and 600 deaths occurred in Foshan (mainland China) in 1978 and in Hainan Province in 1980 and 1986 [27]. These epidemics were characterized by their sudden onset and rapid transmission. Since the 1990s, dengue epidemics have spread gradually from the south-eastern coastal regions to the northern and western regions of China and have become endemic in the south-eastern coastal regions and sporadic in the inner land.

In the present study, we found that DENV-1 was the dominant serotype in Guangzhou, and DENV-2 was the dominant serotype in Xishuangbanna during the dengue outbreaks in 2014–2015 (Fig 2). The DENV-1 viruses circulating during the Guangzhou outbreak were homologous, sharing similar gene sequences not only with the strains previously isolated in Guangdong but also with the Indian strains from the 2009–2011, which suggests that this outbreak was imported (Fig 7) and then circulated in this region. The DENV-1 circulating in Xishuangbanna differs from that in Guangdong but shares similarities with Vietnamese strains, which suggests that this strain was imported from a Southeast Asian country, a finding consistent with the results from a previous study [33]. In contrast, the DENV-2 isolates from this outbreak were heterogeneous and shared similarities not only with the isolates reported by others in this Province but also with the Vietnamese strains [21].

We observed that the viremia levels were higher among patients who tested negative for IgM antibodies compared with the IgM positive ones. The presence of DENV specific IgM antibodies may help with virion clearance through their uptake by phagocytes, a possibility consistent with the results from a previous study which indicated that IgM promote the clearance and limit dissemination of pathogens [34]. We also observed that the viremia level was significantly higher among patients with primary DENV-2 infections (Fig 4B). However, no such difference was observed between the primary and secondary DENV-1 infections. It was interesting to find that the viremia level in the DENV-1 positive Guangzhou patients was one log higher than that of the Xishuangbanna patients, suggesting that this difference is genotype-specific or is related to the host genetic background.

In terms of the clinical manifestations of dengue fever in the study participants, some clinical signs and symptoms were specifically associated with the DENV-2 serotype, such as high rates of headache, anorexia, nausea and vomiting, and general debilitation (Table 1). Higher rising rates of alanine and aspartate transaminases were also observed among those infected with DENV-2 and mixed serotype infections containing DENV-2, indicating the possibility of higher inflammatory reaction rates for these infections. This suggests that DENV-2 infections may cause more serious clinical issues than infections with other serotypes. Although previous reports demonstrated different clinical signs and symptoms with serotype-1 and -2 [35,36,37], this inconsistency may be attributed to different genetic backgrounds. However, no obvious associations among the clinical signs and symptoms of the disease and primary or secondary infections were evident (S1 Table). It is possible that our study did not contain enough patients with specific infection types to be able to draw conclusions about whether the clinical presentations of the disease are associated with a specific infection type. However, the result of one study indicated that some primary infections are associated with severe DENV cases [38]. Many factors, such as genetic background, underlying disease state, and immune response diversity may play roles in the pathogenesis of severe dengue fever during a primary infection [39].

In conclusion, during the DENV outbreaks in Guangdong Province and Yunnan in 2014–2015, the dominant serotypes were DENV-1 genotype V and DENV-2 Cosmopolitan genotype, respectively. DENV-2 is particularly associated with severe dengue infections providing new data to better understanding the pathogenic potential of distinct DENV serotypes in human populations. Therefore, elucidating the immune responses to dengue fever and the pathogenic mechanisms used by DENV are important scientific endeavors. Our phylogenetic analysis showed that during the outbreaks in the two Chinese regions the DENV serotypes we detected shared high homology with the isolates previously reported in Guangdong and Yunnan Province and in Southeast Asian countries, indicating the possibility of dengue fever becoming endemic in China, although most research has attributed the presence of dengue fever in China to imported cases [12,40]. However, dengue infection is not a passing problem in China, and controlling it will require the establishment of a rapid detection system, an improved laboratory-based national surveillance system, a better long-term vector control strategy, and development of safe and effective vaccines and therapeutics to combat it.

## Supporting information

S1 Fig Age distributions of dengue cases in the two geographically distinct study areas (Xishuangbanna and Guangdong) of China. (TIF)

S2 Fig Comparative analyses of amino acid substitutions among 4 strains of DENV-2. There were a total of 13 samples of DENV-2 serotype, and all of them came from Yunnan. Nucleotide alignment indicated: 4 isolates were consistent named as DENV-2 (1), 7 isolates were consistent named as DENV-2 (2), 1 isolate named as DENV-2 (3), 1 isolate named as DENV-2(4). The nucleotide sequence consistency (identity position) of 4 groups was 99.8%. When comparing amino acid, the consistency of DENV-2(2), DENV-2(4), and KX577705 was 100%. Amino acid consistency of DENV-2(1) and DENV-2(3) was 100%, but amino acid consistency of DENV-2(2), DENV-2(4), and KX577705 was 98.8%. At the 215th position, DENV-2(1) andDENV-2(3) amino acids were N-asparagine, DENV-2(2), DENV-2(4) and KX577705 were D aspartic acid.
(TIF)

S1 Table Demographics and the clinical signs and symptoms associated with primary vs secondary DENV infections. (DOCX)

S2 Table Phylogenetic analyses of DENV-1 samples reference sequences. (DOCX)

S3 Table Phylogenetic analyses of DENV-2 samples reference sequences. (DOCX)
