# Supplementary figures and images for: Epidemiological and clinical characteristics of Dengue virus outbreaks in two regions of China, 2014 – 2015
Source: PLoS One. 2019 Mar 5;14(3):e0213353. doi: 10.1371/journal.pone.0213353 (PMC6400443; doi:10.1371/journal.pone.0213353)

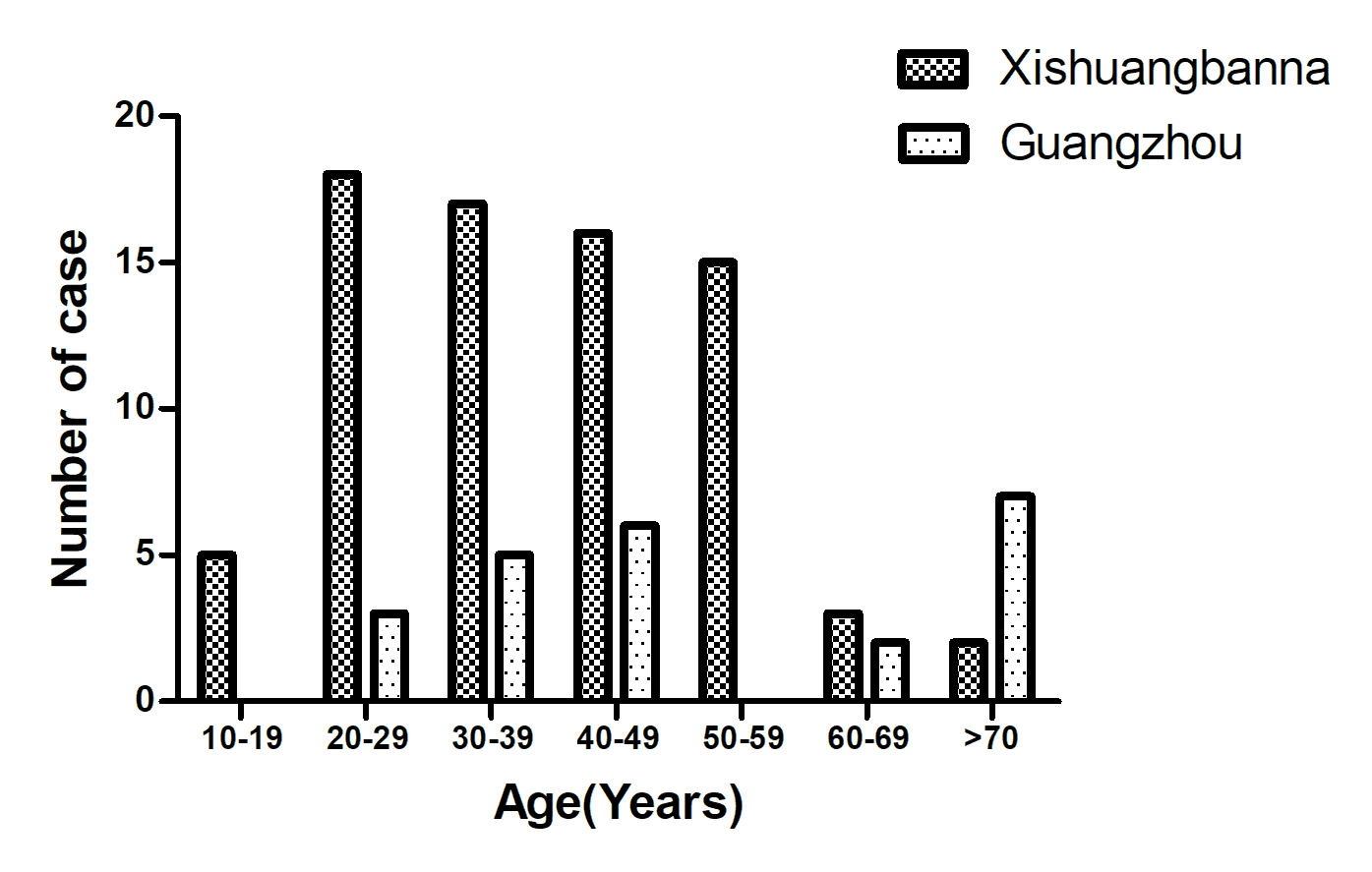

Supplement: S1 Fig — (TIF) [file pone.0213353.s001.tif]

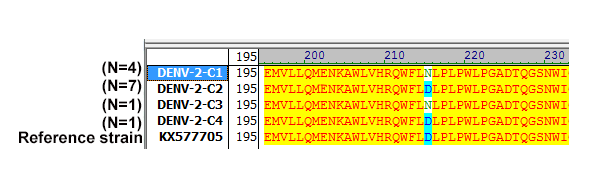

Supplement: S2 Fig — There were a total of 13 samples of DENV-2 serotype, and all of them came from Yunnan. Nucleotide alignment indicated: 4 isolates were consistent named as DENV-2 (1), 7 isolates were consistent named as DENV-2 (2), 1 isolate named as DENV-2 (3), 1 isolate named as DENV-2(4). The nucleotide sequence consistency (identity position) of 4 groups was 99.8%. When comparing amino acid, the consistency of DENV-2(2), DENV-2(4), and KX577705 was 100%. Amino acid consistency of DENV-2(1) and DENV-2(3) was 100%, but amino acid consistency of DENV-2(2), DENV-2(4), and KX577705 was 98.8%. At the 215th position, DENV-2(1) andDENV-2(3) amino acids were N-asparagine, DENV-2(2), DENV-2(4) and KX577705 were D aspartic acid. (TIF) [file pone.0213353.s002.tif]
