# Supplementary material for: Epidemiological and clinical characteristics of Dengue virus outbreaks in two regions of China, 2014 – 2015
Source: PLoS One. 2019 Mar 5;14(3):e0213353. doi: 10.1371/journal.pone.0213353 (PMC6400443; doi:10.1371/journal.pone.0213353)
Supplement: S1 Table — (DOCX) [file pone.0213353.s003.docx]

**S1 Table.Demographics and the clinical signs and symptoms associated with primary vs secondary DENV infections**

| **Infection status （N=57）** | **Total (N=57)** | **Primary infection (N=24)** | **Secondary infection (N=33)** | **P** |
| --- | --- | --- | --- | --- |
| **Age in years,mean（SD）** | 40.45(16.09) | 39.17(13.01) | 41.39(18.96) | 0.596 |
| **Gender,% female** | 31(54.39%) | 15(62.5%) | 16(48.48%) | 0.294 |
| **Fever in the last 7days** | 49(85.96%) | 22(91.66%) | 27(81.82%) | 0.291 |
| **Occupation(%)** | | | | |
| **Unemployed/ retired** | 15(26.32%) | 6(25%） | 9(37.5%) | ND |
| **Laborer** | 15(26.32%) | 4(16.67%) | 11(33.33%) | ND |
| **Merchant/office** | 20(35.09%) | 11(45.83%) | 9(37.5%) | ND |
| **Other** | 7(12.28%) | 3(12.5%) | 4(12.12%) | ND |
| **Clinical sign and symptoms(%)** | | | | |
| **Headache** | 37(64.91%) | 17(70.83%) | 20(60.61%) | 0.424 |
| **Cough** | 10(17.54%) | 4(16.67%) | 6(18.18%) | 0.882 |
| **Anorexia and nausea** | 30(52.63%) | 14(58.33%) | 16(48.48%) | 0.536 |
| **Muscle/joint pain** | 40(70.18%) | 19(79.17%) | 21(63.64%) | 0.206 |
| **Rash** | 19(33.33%) | 7(29.17%) | 12(36.36%) | 0.569 |
| **Bleeding** | 12(21.05%) | 2(8.33%) | 10(30.30%) | **0.045** |
| **Vomiting** | 17(29.82%) | 6(25%) | 11(33.33%) | 0.487 |
| **Debilitation** | 25(43.86%) | 12(50%) | 13(39.39%) | 0.426 |
| **Abdominal pain** | 2(3.51%) | 2(8.33%) | 0 | 0.091 |
| **Diarrhea** | 10(17.54%) | 6(25%) | 4(12.12%) | 0.207 |
| **Main laboratory results during the course of the disease(%)** | | | | |
| **Thrombocytopenta（<100×10^9^/L）** | 38(66.67%) | 16(66.67%) | 22(66.67%) | 1 |
| **Leukopenia（<4×10^9^/L）** | 39(68.42%) | 16(66.67%) | 23(69.70%) | 0.808 |
| **ALT(>40U/L)** | 51(89.47%) | 23(95.83%) | 28(84.85%) | 0.182 |
| **AST(>40U/L)** | 52(91.23%) | 22(91.67%) | 30(90.91%) | 0.902 |

*ND*:Nodate
