# Supplementary material for: Epidemiological and clinical characteristics of Dengue virus outbreaks in two regions of China, 2014 – 2015
Source: PLoS One. 2019 Mar 5;14(3):e0213353. doi: 10.1371/journal.pone.0213353 (PMC6400443; doi:10.1371/journal.pone.0213353)
Supplement: S2 Table — (DOCX) [file pone.0213353.s004.docx]

**S2 Table. Phylogenetic analyses of DENV-1 samples reference sequences**

| **Serotype** | **Genotype** | **Aceesion No.** | **Location** | **Year** |
| --- | --- | --- | --- | --- |
| DENV-1 | Ⅰ | AF350498 | Guangzhou China | 1980 |
| DENV-1 | Ⅰ | AY732477 | Thailand | 1991 |
| DENV-1 | Ⅰ | AF298808 | Djibouti | 1998 |
| DENV-1 | Ⅰ | DQ193572 | Fujian China | 2004 |
| DENV-1 | Ⅰ | AY835999 | Zhejiang China | 2004 |
| DENV-1 | Ⅰ | EU081260 | Singapore | 2005 |
| DENV-1 | Ⅰ | HM181939 | Cambodia | 2006 |
| DENV-1 | Ⅰ | EU249494 | Vietnam | 2006 |
| DENV-1 | Ⅰ | GQ868632 | Cambodia | 2008 |
| DENV-1 | Ⅰ | GQ868615 | Vietnam | 2008 |
| DENV-1 | Ⅰ | KJ545444 | Guangdong China | 2013 |
| DENV-1 | Ⅰ | KX056459 | Yunnan China | 2015 |
| DENV-1 | Ⅱ | AF425629 | Thailand | 1963 |
| DENV-1 | Ⅱ | AF180817 | Thailand | 1964 |
| DENV-1 | Ⅲ | AF425622 | Myanmar | 1972 |
| DENV-1 | Ⅲ | EF457905 | Myanmar | 1972 |
| DENV-1 | Ⅲ | FN825674 | Myanmar | 2005 |
| DENV-1 | Ⅳ | EF025110 | Guangzhou China | 1971 |
| DENV-1 | Ⅳ | FJ196845 | Guangzhou China | 1991 |
| DENV-1 | Ⅳ | EF032590 | Guangzhou China | 1995 |
| DENV-1 | Ⅳ | AB189120 | Indonesia | 1998 |
| DENV-1 | Ⅳ | AB195673 | Japan | 2003 |
| DENV-1 | Ⅳ | AB204803 | Japan | 2004 |
| DENV-1 | Ⅴ | AY713473 | Myanmar | 1971 |
| DENV-1 | Ⅴ | AY732474 | Thailand | 1980 |
| DENV-1 | Ⅴ | AF298807 | Coate d'Ivoire | 1998 |
| DENV-1 | Ⅴ | DQ285562 | Comoros | 1993 |
| DENV-1 | Ⅴ | AY762084 | Singapore | 1993 |
| DENV-1 | Ⅴ | M87512 | Singapore | 1993 |
| DENV-1 | Ⅴ | AY722803 | Myanmar | 1998 |
| DENV-1 | Ⅴ | GQ357692 | Singapore | 2008 |
| DENV-1 | Ⅴ | JN903579 | India | 2008 |
| DENV-1 | Ⅴ | JQ917404 | India | 2009 |
| DENV-1 | Ⅴ | JQ692085 | India | 2010 |
| DENV-1 | Ⅴ | KF289072 | India | 2011 |
| DENV-1 | Ⅴ | KT827377 | Guangzhou China | 2014 |
| DENV-2 |  | NC001474 | Thailand | 1964 |
| DENV-2 |  | EF105386 | Burkina Faso | 1980 |
| DENV-2 |  | EF105378 | Guinea | 1981 |
| DENV-2 |  | GQ199892 | Jamaica | 2007 |
| DENV-3 |  | AY676353 | Thailand | 1987 |
| DENV-3 |  | FJ898455 | Cook Islands | 1991 |
| DENV-3 |  | FJ410176 | Puerto Rico USA | 2000 |
| DENV-3 |  | NC001475 | Sri Lanka | 2000 |
| DENV-4 |  | AF326573 | Dominica Island | 1981 |
| DENV-4 |  | FJ196850 | Guangdong China | 1990 |
| DENV-4 |  | AY618990 | Bangkok Thailand | 1991 |
| DENV-4 |  | GQ398256 | Singapore | 2005 |
| DENV-1 |  | Sample | Guangzhou China | 2014 |
| DENV-1 |  | Sample | Yunnan China | 2015 |
