# Supplementary material for: Epidemiological and clinical characteristics of Dengue virus outbreaks in two regions of China, 2014 – 2015
Source: PLoS One. 2019 Mar 5;14(3):e0213353. doi: 10.1371/journal.pone.0213353 (PMC6400443; doi:10.1371/journal.pone.0213353)
Supplement: S3 Table — (DOCX) [file pone.0213353.s005.docx]

**S3 Table. Phylogenetic analyses of DENV-2 samples reference sequences**

| **Serotype** | **Genotype** | **Aceesion No.** | **Location** | **Year** |
| --- | --- | --- | --- | --- |
| DENV-2 | American | M19197 | Puerto Rico | 1969 |
| DENV-2 | American | AF100465 | Venezuela | 1987 |
| DENV-2 | American | AF100469 | Mexicom | 1992 |
| DENV-2 | American/Asian | M20558 | Jamaica | 1983 |
| DENV-2 | American/Asian | AF119661 | Hainan China | 1985 |
| DENV-2 | American/Asian | DQ181801 | Thailand | 1990 |
| DENV-2 | American/Asian | AF410367 | Vietnam | 1998 |
| DENV-2 | American/Asian | AF489932 | Brazil | 1998 |
| DENV-2 | American/Asian | GU434159 | VietNam | 2004 |
| DENV-2 | American/Asian | JX051813 | Paraguay | 2010 |
| DENV-2 | AsianⅠ | AF264053 | Thailand | 1980 |
| DENV-2 | AsianⅠ | U87411 | Thailand | 1984 |
| DENV-2 | AsianⅠ | AF100462 | Thailand | 1995 |
| DENV-2 | AsianⅠ | HQ588138 | Vietnam | 2006 |
| DENV-2 | AsianⅠ | JX101615 | Vietnam | 2011 |
| DENV-2 | AsianⅠ | KF060920 | Guangzhou China | 2013 |
| DENV-2 | AsianⅠ | KJ939398 | China | 2013 |
| DENV-2 | AsianⅡ | KC812278 | Taiwan | 1987 |
| DENV-2 | AsianⅡ | AF204178 | GuangxiChina | 1987 |
| DENV-2 | AsianⅡ | AF204177 | Hainan China | 1989 |
| DENV-2 | Cosmopolita | AB111448 | Indonesia | 1994 |
| DENV-2 | Cosmopolita | AB189123 | Indonesia | 1998 |
| DENV-2 | Cosmopolita | AB189122 | Indonesia | 1998 |
| DENV-2 | Cosmopolita | EF051521 | Zhongshan China | 2001 |
| DENV-2 | Cosmopolita | AY858036 | Indonesia | 2004 |
| DENV-2 | Cosmopolita | EU179857 | Brunei | 2005 |
| DENV-2 | Cosmopolita | EU081177 | Singapore | 2005 |
| DENV-2 | Cosmopolita | AB219135 | Easttimor | 2005 |
| DENV-2 | Cosmopolita | EU482640 | Vietnam | 2006 |
| DENV-2 | Cosmopolita | KC762675 | Indonesia | 2008 |
| DENV-2 | Cosmopolita | KC762678 | Indonesia | 2010 |
| DENV-2 | Cosmopolita | JN009091 | Guangzhou China | 2010 |
| DENV-2 | Cosmopolita | JN568243 | Indonesia | 2010 |
| DENV-2 | Cosmopolita | JN544399 | Singapores | 2011 |
| DENV-2 | Cosmopolita | JF968050 | Taiwan | 2012 |
| DENV-2 | Cosmopolita | KF052650 | Inodonesia | 2012 |
| DENV-2 | Cosmopolita | KT175129 | Vietnam | 2014 |
| DENV-2 | Cosmopolita | KY971741 | Vietnam | 2015 |
| DENV-2 | Cosmopolita | KX262952 | Yunnan China | 2015 |
| DENV-2 | Cosmopolita | KX577705 | Yunnan China | 2015 |
| DENV-2 | Sylvatic | AF231717 | Malysia | 1970 |
| DENV-1 |  | KT827377 | Guangzhou China | 2014 |
| DENV-1 |  | FJ639692 | Cambodia | 2007 |
| DENV-1 |  | GQ868632 | Cambodia | 2008 |
| DENV-1 |  | KX056459 | Yunnan China | 2015 |
| DENV-3 |  | AY676353 | Thailand | 1987 |
| DENV-3 |  | FJ898455 | Cook Islands | 1991 |
| DENV-3 |  | NC001475 | Sri Lanka | 2000 |
| DENV-4 |  | FJ196850 | Guangdong China | 1990 |
| DENV-4 |  | AY618990 | Thailand | 1991 |
| DENV-4 |  | GQ398256 | Singapore | 2005 |
| DENV-2 |  | Sample | Yunnan China（1） | 2015 |
| DENV-2 |  | Sample | Yunnan China（2） | 2015 |
| DENV-2 |  | Sample | Yunnan China（3） | 2015 |
| DENV-2 |  | Sample | Yunnan China（4） | 2015 |
